# Effect of Immersive Virtual Reality Teamwork Training on Safety Behaviors During Surgical Cases: Nonrandomized Intervention Versus Controlled Pilot Study

**DOI:** 10.2196/66186

**Published:** 2025-05-01

**Authors:** Lukasz Mazur, Logan Butler, Cody Mitchell, Shaian Lashani, Shawna Buchanan, Christi Fenison, Karthik Adapa, Xianming Tan, Selina An, Jin Ra

**Affiliations:** 1 Department of Radiation Oncology, Division of Healthcare Engineering, School of Medicine, University of North Carolina, Campus Box 7512, Chapel Hill, NC, 27515, United States, 1 9196169702; 2 School of Information and Library Science, University of North Carolina, Chapel Hil, NC, United States; 3 Gillings School of Global Public Health, University of North Carolina, Biostatistics, Chapel Hill, NC, United States; 4 Department of Surgery, School of Medicine, University of North Carolina, Chapel Hill, NC, United States

**Keywords:** Teamwork Evaluation of Non-Technical Skills, TENTS, Team Strategies and Tools to Enhance Performance and Patient Safety, TeamSTEPPS, immersive virtual reality, virtual reality, VR, safety behavior, surgical error, operating room, OR, training intervention, training, pilot study, nontechnical skills, surgery, surgical, patient safety, medical training, medical education

## Abstract

**Background:**

Approximately 4000 preventable surgical errors occur per year in the US operating rooms, many due to suboptimal teamwork and safety behaviors. Such errors can result in temporary or permanent harm to patients, including physical injury, emotional distress, or even death, and can also adversely affect care providers, often referred to as the “second victim.”

**Objective:**

Given the persistence of adverse events in the operating rooms, the objective of this study was to quantify the effect of an innovative and immersive virtual reality (VR)–based educational intervention on (1) safety behaviors of surgeons in the operating rooms and (2) sense-making regarding the overall training experience.

**Methods:**

This mixed methods pre- versus postintervention pilot study was conducted in a large academic medical center with 55 operating rooms. Safety behaviors were observed and quantified using validated Teamwork Evaluation of Non-Technical Skills instrument during surgical cases at baseline (101 observations; 83 surgeons) and postimmersive VR based intervention (postintervention: 24 observations within each group; intervention group [with VR training; 10 surgeons] and control [no VR training; 10 surgeons]). VR intervention included a 45-minute immersive VR-based training incorporating a pre- and postdebriefing based on Team Strategies and Tools to Enhance Performance and Patient Safety (TeamSTEPPS) principles to improve safety behaviors. A 2-tailed, 2-sample *t*-test with adjustments for multiplicity of the tests was used to test for significance in observable safety behaviors between the groupings. The debriefing data underwent analysis through the phenomenological analysis method to gain insights into how participants interpreted the training.

**Results:**

Preintervention, all safety behaviors averaged slightly above “acceptable” scores, with an overall average of 2.2 (range 2‐2.3; 0‐3 scale). The 10 surgeons that underwent our intervention showed statistically significant (*P*<.05) improvements in 90% (18/20) of safety behaviors when compared to the 10 surgeons that did not receive the intervention (overall average 2.5, range 2.3‐2.7 vs overall average 2.1, range 1.9‐2.2). Our qualitative analysis based on 492 quotes from participants suggests that the observed behavioral changes are a result of an immersive experience and sense-making of key TeamSTEPPS training concepts.

**Conclusions:**

VR-based immersive training intervention focused on TeamSTEPPS principles seems effective in improving safety behaviors in the operating rooms as quantified via observations using the Teamwork Evaluation of Non-Technical Skills instrument. Further research with larger, more diverse sample sizes is needed to confirm the generalizability of these findings.

## Introduction

High-quality health care necessitates ongoing efforts to reduce the occurrence of medical errors [1]. Surgical patients face heightened risks of adverse outcomes related to errors due to the invasive nature of surgical procedures [2]. It is estimated that more than 4000 preventable surgical errors occur annually on a national scale [1,2]. Such errors can result in temporary or permanent harm to patients, including physical injury, emotional distress, or even death, and can also adversely affect care providers, often referred to as the “second victim.” A notable example is the unintended retention of foreign objects, which is believed to happen at least once in every 5500 surgeries [3]. This can lead to the need for reoperation, extended hospital stays, and complications such as sepsis. Furthermore, the average additional cost associated with each incident of unintended retention is estimated to exceed US $200,000 [4]. Common underlying causes of surgical errors identified by the Joint Commission include the lack of established policies and procedures, issues related to hierarchy and intimidation, ineffective communication among care team, and the failure of staff to relay pertinent patient information [5]. Additionally, factors such as excessive workload, time constraints, and burnout are linked to increased error rates [5]. Addressing these root causes has proven challenging, as complex health care delivery systems tend to evolve over time, leading to the emergence of new failure sources and pathways. Teamwork skills are often essential for preventing such errors that could lead to patient harm [6]. [7], To address these issues, the Team Strategies and Tools to Enhance Performance and Patient Safety (TeamSTEPPS) framework was specifically designed as a resource to help health care providers improve patient safety behaviors through effective communication, leadership, situation monitoring, and mutual support [8]. [9,10] By using the TeamSTEPPS framework, it is possible to assess the use and quality of patient safety education and behaviors among operating room staff and establish a baseline for improvement.

Virtual reality (VR) is a digital technology that enables a virtual manifestation of the real world [11]. VR provides a more captivating experience compared to viewing a conventional video, as it fully envelopes the viewer within the narrative [12]. In VR, the audience becomes an integral part of the story rather than merely an onlooker. The advantages of immersive VR primarily include: (1) viewers are placed within a 360-degree environment, where each movement of the head unveils new dimensions of the scene. Conversely, traditional video confines viewers to a fixed perspective on a flat screen. (2) Participants in VR actively engage with the narrative, rather than remaining passive spectators. (3) A profound experiential learning opportunity, allowing participants to engage with the contextual realities of a surgical error in the surgical environment.

While live mock simulations with standardized patients are used for certain health care scenarios, the logistical demands of accurately recreating a surgical environment are substantial. These include the coordination of a full surgical team, time spent in an operating room, and the creation of a realistic setting, along with patient representation and various special effects that must align with the error, necessitating cleanup and reset after each simulation. The extensive resources required make this approach neither cost-effective nor scalable for providing multiple realistic experiences for individual learners. Furthermore, a live mock simulation often fails to address the comprehensive needs of learners, as it does not provide insights into the broader contributing factors and repercussions that extend beyond the confines of the operating room. While this disconnect may not significantly affect certain types of learning, such as factual recall, the deeper cognitive processing of theoretical scenarios, particularly in the context of complex situations, could be enhanced through more experiential learning methods such as VR [13]. Experiential learning fosters a personalized and cognitive interaction with educational content, highlighting the relationship between learning and its practical application in the real world. If health care workers are to engage in sense-making regarding the intricate realities associated with adverse events, VR may provide distinct advantages over 2D content and live mock simulations by offering an immersive perspective of events as they would unfold in real life.

In recent years, VR has seen increasing use for safety training across industries [14-17]. VR safety training has great potential, as it allows trainees to experience complex, challenging situations that are difficult to replicate in the real world due to ethical, cost, and time constraints. However, there is a lack of research examining the efficacy of immersive VR-based interventions focused on safety education and behaviors as proposed by the TeamSTEPPS framework. Thus, given the persistence of adverse events in the operating rooms, the objective of this pilot study was to quantify the effect of an innovative and immersive VR-based educational intervention focused on TeamSTEPPS on (1) safety behaviors of surgeons in the operating rooms and (2) sense-making regarding the overall training experience and contributing factors associated with the surgical error.

## Methods

### Ethical Considerations

This study obtained ethics approval via the Institutional Review Board of the University of North Carolina at Chapel Hill (22‐1150). Participants were provided with and signed an informed consent form before engaging with activities related to this study. Participants in the intervention group were provided with a small token of appreciation ($25) for compleating the VR intervention process. Patients or the public were not involved in the design, conduct, reporting, or dissemination plans of our research. All results are reported in an aggregate manner to ensure the privacy and confidentiality of participants. The protocol for the full mixed methods pre- versus postintervention study design was published in *JMIR Research Protocols* [18].

### Recruitment

A scripted email with a flyer was sent through a listserv to inform prospective participants about this study. Our research team was on standby to answer any questions from prospective participants, and the principal investigators’ contact information was available on the flyer. First, for the baseline measurement, a volunteer sample of 83 surgeons (35 attendings, 41 residents, and 7 fellows; 36 female and 47 male) was enrolled and participated. Surgeons volunteered by providing verbal consent to be observed and scored for safety behaviors before each surgical case. One surgeon refused to participate (no reasons stated). For the pilot study, 10 out of 83 (12%) surgeons (7 attendings and 3 residents; 5 female and 5 male) volunteered to undergo the immersive VR-based educational intervention and to undergo the follow-up observations and scoring. An additional 10 of 83 (12%) surgeons (10 attendings and 0 residents; 3 female and 7 male) volunteered to undergo follow-up observation and scoring without being exposed to the intervention.

### Study Design

This mixed methods pre- versus postintervention study with a baseline and intervention or control groups was conducted at a large academic medical center with 55 operating rooms. For the baseline measurement, safety behaviors were quantified while observing 101 surgical cases from October 31, 2022, to February 21, 2023, with 83 surgeons. For the pilot study, 24 observations within each group were conducted from April 17, 2023, to November 2, 2023, with 10 surgeons in the intervention and control group, respectively. Data was collected using the Teamwork Evaluation of Non-Technical Skills (TENTS) instrument. We opted for the TENTS instrument instead of alternatives such as the TeamSTEPPS Team Performance Observation Tool due to our requirement for a more focused evaluation of nontechnical teamwork aspects. TENTS is specifically designed to assess nontechnical dimensions of teamwork, encompassing team member interactions, task delegation, and information management, which makes it particularly relevant in situations where technical skills are not the primary concern. Additionally, TENTS provides a more detailed observational framework, allowing for a comprehensive evaluation of specific teamwork behaviors, in contrast to the broader categories covered by the TeamSTEPPS Team Performance Observation Tool. In our study, we used TENTS items 1A to 4D to assess individual behaviors of surgeons in independent settings (except for the 3 residents who were part of the intervention group), while items 5 and 6 were used to evaluate the overall team functioning and leadership. To train the observers for scoring items 5 and 6, we assembled a team of 17 medical student volunteers who were tasked with conducting observations using the TENTS instrument. They used a simplified scoring scale ranging from 0 to 3, where 0 indicates expected behavior not observed, 1 signifies observed behavior that was poorly executed or counterproductive, 2 denotes acceptable performance, and 3 represents excellent performance. The students received 1.5 hours of instruction on TeamSTEPPS patient safety behaviors and were trained to apply the validated TENTS instrument consistently. Following this, they participated in facility tours and were guided through the observation protocol, which included a practical demonstration of the TENTS scoring process. The students were assigned to surgical cases by aligning their weekly schedules with the relevant cases, while being blinded to the treatment status of the surgeons during postintervention observations. TENTS scores were recorded during real-time observations of surgical procedures using paper forms, and students were required to provide written justifications for behaviors rated as 1 (poor) or 3 (excellent) to offer context for their evaluations.

### Interventions or Exposures

We used state-of-the-art filming equipment to capture a 360° view of the event and the perspective of those involved in the error event and contributing events. We built the scripts for the scenes, recruited actors (attendings, residents, students, and administrators) with lived experiences in health care to help with the filming, identified filming locations, rehearsed all the scenes, and filmed our scenes. This training was delivered to the participants using a VR head-mounted display to ensure an immersive environment (see Multimedia Appendix 1 for a 1-minute summary clip of the training). Specifically, we used the Pico Neo 3 Pro Eye headset (PICO Technology Co Ltd) with 6DoF VR hardware or software to administer the training.

Participants were exposed to a 45-minute immersive VR-based training based on TeamSTEPPS principles to improve safety behaviors. Overall, our overarching training story is focused on a human error that occurs at the operating table but is caused by many factors, as explained by the James Reason Swiss Cheese Model [19] and Human Factors Analysis and Classification System (HFACS) [20]. Training involved a standardized pre- and postbriefing aimed at comprehending and identifying potential behavioral enhancements within the training narrative, grounded in TeamSTEPPS principles. Specifically, we sought to collect participants’ perspectives on the overall training experience and their interpretation of the TENTS and HFACS-related factors that contributed to the patient safety incident illustrated in the VR training. The debriefing data underwent analysis through the phenomenological analysis method [21] to gain insights into how participants interpreted the training and the patient safety incidents they encountered, thereby refining their understanding, behavior, and commitment to TeamSTEPPS practices. This qualitative analysis was performed by 2 researchers, a senior surgical attending and a fourth-year surgical resident, using the data frame theory of sense-making [22]. The primary objective of this qualitative effort was to enhance our understanding of the main outcomes and measures outlined below.

### Main Outcomes and Measures

The primary outcome measures were the pooled average and 95% CI of observed TeamSTEPPS related behaviors quantified using the validated TENTS instrument (including 20 types of safety behaviors across 4 domains [communication, leadership, situation monitoring, and mutual support] scored from 0=expected but not observed, 1=observed but poorly performed or counterproductive, 2=observed and acceptable, and 3=observed and excellent).

### Statistical Analysis

A 2-tailed, ANOVA was used to test for significance between the groupings. For given comparators (eg, [1] vs [2] or [2] vs [3]), we used a false discovery rate (FDR) control (the Benjamini-Hochberg procedure) to adjust the resulting *P* values (from the 2-sample *t*-tests) to account for the multiplicity of the tests. We claimed a result to be statistically significant if the adjusted *P* value is less than .05. All analyses were performed with R statistical software. Data analysis was conducted from August 10 to December 1, 2023.

## Results

Preintervention, all safety behaviors averaged slightly above 2 (“acceptable”), with an overall average of 2.2 (range 2‐2.3). There was no significant difference between the intervention and the control group at the preintervention stage. The results indicated that the 10 surgeons that underwent our intervention showed statistically significant (*P*<.05) improvements in 90% (18/20) of safety behaviors when compared to the 10 surgeons that did not receive the intervention (overall average 2.5, range 2.3‐2.7 vs overall average 2.1, range 1.9‐2.2; Table 1). Our qualitative analysis revealed 492 individual quotes. The results suggest that the observed behavioral changes are a result of a sense-making emerging from 5 specific themes as discussed below.

**Table 1.** Summary of results (baseline vs intervention).

	TENTS^a^ behavior	Baseline, mean (SD)	Intervention, mean (SD)	*P* value
1a	Communicates and receives information appropriately	2.227 (0.53)	2.708 (0.455)	.001
1b	Comfortable speaking up and asking questions	2.237 (0.452)	2.625 (0.484)	.001
1c	Responses to feedback between team members	2.135 (0.504)	2.458 (0.498)	.02
1d	Communicates and receives information to or from patient	2.097 (0.433)	2.261 (0.439)	.14
1e	Uses language in urgent situations appropriately	2.133 (0.505)	2.444 (0.497)	.04
1f	Uses teamwork tools	2.26 (0.464)	2.542 (0.498)	.03
1g	Learns together, focuses on improvement following a problem	2.222 (0.451)	2.55 (0.497)	.02
2a	Leaders effectively manage team during their roles	2.274 (0.493)	2.708 (0.455)	.001
2b	Verbalizes plan: intentions, recommendations, or timeframes	2.247 (0.501)	2.583 (0.571)	.02
2c	Delegates tasks appropriately	2.104 (0.369)	2.458 (0.498)	.007
2d	Instructs as appropriate to the situation	2.281 (0.475)	2.542 (0.498)	.03
3a	Pays attention to surroundings or environment	2.11 (0.526)	2.5 (0.5)	.005
3b	Aware of each other, contributions, strengths, and weaknesses	2.208 (0.433)	2.5 (0.5)	.02
3c	Verbalizes adjustments in plan as changes occur	2.236 (0.428)	2.524 (0.499)	.03
4a	Willingness to ask for help or additional resources	2.26 (0.464)	2.708 (0.455)	.001
4b	Willingness to support others across different roles	2.253 (0.437)	2.417 (0.493)	.15
4c	Accomplishes and prioritizes tasks appropriately	2.103 (0.338)	2.333 (0.471)	.04
4d	Employs conflict resolution	2.038 (0.341)	2.316 (0.465)	0.03
5	Rating of how well the team functioned as a whole	2.258 (0.44)	2.708 (0.455)	0.001
6	Rate how well leaders functioned and how the team responded	2.247 (0.434)	2.708 (0.455)	0.001

a TENTS: Teamwork Evaluation of Non-Technical Skills.

## Discussion

### Principal Results

#### Overview

The results show that participants exposed to our intervention displayed improved levels of safety behaviors, as quantified by the TENTS instrument, in 90% (18/20) of the safety behaviors measured. Specifically, quantitative data suggests that surgical teams were more effective in developing and maintaining a dynamic awareness of the situation in the operating room. This was achieved by assembling and understanding data from various sources (eg, patient, team, time, displays, and equipment), and using strong communication and leadership skills to think ahead and provide clear direction while being considerate of individual team members’ needs. Importantly, these improvements were observed not only at the individual level, as shown by the TENTS instrument, but also in the overall team functioning and leadership, as indicated by aggregate measures of “how well the team functioned as a whole,” ”how well the leaders functioned,” and “how the team responded.”

The 2 behaviors that did not reach statistical significance, despite trending positively, were 1d (communicating and receiving information with patients), and 4b (willingness to support others across different roles) (Table 1). For the communication behavior, very few such interactions were observed during the surgical cases, limiting our assessment of this behavior. Regarding the willingness to support others across roles, this was the highest-scoring behavior at baseline, suggesting it may have been challenging to improve further. This implies that in the dynamic and complex surgical environment, team members may struggle to step outside their designated roles to provide support to one another, as they focus intently on delivering excellent patient care and ensuring safety within their specific responsibilities.

Our qualitative analysis suggests that these behavioral improvements materialized from the enhanced understanding of skills needed in the operating room by reinforcing critical behaviors related to the sense-making of themes presented below.

#### Need for Effective Teamwork and Communication

The VR training modeled effective versus ineffective team communication, demonstrating how dismissing or ignoring concerns can lead to errors and decrease team trust. Participants may have practiced assertive communication, such as how a resident can escalate a concern when an anesthesiologist is distracted or how surgical technology can improve instrument handling. VR also emphasized calling out errors in a constructive way, rather than scolding or ignoring them.

The most consistent topics addressed by the subjects during the poststudy interview were teamwork and communication, and how these traits are essential for a well-functioning operating room. Participants noted how the VR training vividly illustrated both the consequences of poor communication and the benefits of a cohesive team. The training emphasized that errors often arise not solely from technical mistakes but from an inability to effectively relay and escalate concerns. The lack of teamwork and communication in the ineffective VR scenario was particularly alarming to participants. One individual highlighted the disconnect among team members: “There was clearly not a deep relationship between the surgeon, the resident, the scrub, the circulator, and the anesthesiologist; they just seemed completely disconnected [and] in their own worlds.” Another key issue was the absence of assertive communication when concerns arose. One participant recalled: “The resident did pick up on the change in the heart rate tone, questioned the anesthesiologist about it, who blew her off.” The absence of closed-loop communication was another prevalent theme. Participants remarked on how essential feedback loops were often missing in the ineffective scenarios, which contributed to preventable errors: “No closed-loop communication. Just a lot of people suggesting things but the other person either wasn’t listening or just kind of ignored it and moved along. Even when it was something that could have prevented safety issues.” Overall, the poststudy interviews reinforced that teamwork and communication are foundational to operating room effectiveness. The VR training provided a powerful demonstration of how dismissing concerns, failing to engage in open dialogue, and neglecting structured communication can significantly hinder patient safety. By recognizing these pitfalls and emphasizing assertive, closed-loop communication, participants reported a newfound appreciation for fostering a more cohesive and communicative operating room team.

#### Emphasis on Empathetic Workplace Culture and Psychological Safety

VR heightened participants’ awareness of how fatigue, dismissive communication, and team dynamics impact psychological safety in the operating room. By simulating real-world scenarios, it demonstrated how exhaustion and distractions compromise decision-making, how seemingly minor quips or sarcasm can erode team cohesion, and how validating trainee concerns fosters a culture of safety and respect. This reinforced the importance of clear, professional communication and proactive leadership in creating a supportive and effective surgical intervention.

The VR training underscored how the psychological safe environment of the operating room directly impacts team effectiveness, patient safety, and overall workplace culture. Participants became more empathetic and attuned to how fatigue, dismissive behavior, and team dynamics can create a toxic versus supportive surgical setting. By immersing participants in scenarios where exhaustion led to oversight, sarcasm eroded trust, and concerns were either validated or dismissed, the training highlighted the importance of maintaining a psychologically safe and healthy work environment. One of the most striking realizations was how fatigue and distraction—often seen as inevitable in surgical practice—could significantly impair decision-making and communication. As one participant noted, “Exhaustion, lack of sleep, and lack of focus on the attending’s part... distraction from the anesthesiologist... these are indicators that their wellness score is probably not stellar.” Additionally, the training revealed how subtle, seemingly harmless behaviors can undermine psychological safety. Participants observed how sarcastic remarks or casual quips, even when meant humorously, created an environment where individuals felt less comfortable speaking up. “There were a lot of quips...I don’t think they contributed too much. And they can be detrimental.” Another crucial takeaway was the need to legitimize concerns when raised, rather than allowing the pressures of a high caseload to override safety. One participant reflected, “I think we can always do better legitimizing people raising concerns...the pressure to feel rushed and move quickly ... can obviously be counterproductive.” Perhaps most telling was the recognition that team dynamics set the tone for the entire operating room. When interpersonal relationships are strained, it affects everyone, from the attending surgeon to the anesthesia technician. One participant encapsulated this sentiment: “Because I think we’ve all been in rooms where [if] the staff doesn’t get along …it makes it miserable for everyone.” The VR training effectively demonstrated how the psychological environment of the operating room shapes both patient safety and team dynamics, making participants more aware of the subtle but powerful ways fatigue, communication styles, and validation of concerns impact surgical outcomes. By immersing participants in realistic scenarios, the training seemed to inspire an appreciation for operating rooms, where they do have a psychologically healthy environment.

#### Need for Leadership With Personal Responsibility and Accountability

VR simulation highlighted the ripple effect of individual actions—how fatigue, inattention, or lack of engagement from a team member impacts the whole operating room. It also reinforced that leaders (attendings, residents, or nurses) set the tone for safety culture, whether by ensuring protocols are followed or by fostering an environment where concerns can be raised. The VR training may have helped participants recognize their personal accountability in maintaining operating room safety and identifying behaviors that contribute to or undermine team effectiveness.

The training reinforced the critical role that leadership plays in shaping operating room culture, particularly in fostering accountability and ensuring adherence to safety protocols. Participants observed how leadership—or the lack thereof—had a cascading effect on communication, decision-making, and overall team cohesion. By placing participants in scenarios where leadership failures led to errors or unsafe practices, the training emphasized the responsibility of every operating room member to contribute to a culture of accountability. A key takeaway was the attending surgeon’s responsibility in setting the tone for the operating room environment. As 1 participant remarked, “The attending surgeon sort of sets the tone in many ways, and by not looking into concerns...[and] cutting corners in order to be able to increase throughput...—that’s just not good leadership.” Beyond the attending, participants recognized how personal accountability extends to every team member. The training exposed moments where concerns were voiced quietly but never formally escalated, leading to missed opportunities to address potential safety issues. One participant reflected, “...everyone was making little comments, but no one was, again, like really saying them. They were all kind of talking to themselves....” The VR also made participants more aware of how the pressure to move quickly can lead to cutting corners, potentially compromising patient outcomes. One participant acknowledged, “We all get caught up in rush, rush, rush…and I’ve done that before, where you look through the labs, like, it’s probably fine. It usually is. But what if it’s not?” Ultimately, the training reinforced the idea that each case demands responsibility and accountability. The VR experience demonstrated how a disengaged or inattentive leader could undermine these traits by dismissing concerns or prioritizing efficiency over protocol.

#### Need for Stability

The VR training elicited participants’ desires and personal experiences to have surgical teams that are consistent and connected. Understandably, those who are working in a high-stakes, high-stress environment would want to mitigate other factors that could lead to negative patient outcomes or contribute to workplace burnout and distress. The VR training showed participants what can happen with a more discordant or unstable working environment, which can mimic reality, and many participants were quick to point out how deleterious that can be to the dynamics of an operating room.

The training highlighted the critical role that stability plays in fostering an effective and supportive surgical environment. Participants emphasized the need for consistency in team composition, resource availability, and leadership presence—elements that are often taken for granted but can significantly impact patient safety and staff well-being. In the high-stakes, high-stress environment of the operating room, an unstable or discordant team structure can lead to inefficiencies, miscommunication, and increased burnout, all of which were vividly demonstrated in the VR scenarios. Many participants noted how instability, whether due to staffing shortages, systemic pressures, or administrative constraints, can disrupt operating room dynamics. One participant reflected on the broader hospital structure, stating, “It didn’t seem like there was support from a majority of people...No one person can do it all.” A major recurring theme was the strain placed on attendings who were expected to be in multiple places at once, highlighting the impact of systemic pressures on operating room stability. As 1 participant observed, “I mean, there were clearly systemic pressures for the attending to be in multiple places at one time, I would say primarily pressure for throughput, short staffing.” This speaks to the broader challenges of balancing efficiency with quality care. This particularly resonated with our interviewees as it is something that a vast majority of health care workers can relate to. By experiencing the challenges of an unstable operating room environment with the constant, relatable pressure to do more, participants gained a deeper appreciation for the structures and policies needed to foster a more reliable and effective surgical setting.

#### Emphasis on Outcome and Attention to Detail

This VR module showed participants what can happen when means do not at all justify ends. In a system where outcomes, whether it be several cases completed or the speed of the operation, are prioritized over how those end points are achieved, it can lead to consequential errors. While operating rooms need to maintain a high level of efficiency, the consequences of doing so can come at the expense of the patient. It can be challenging for surgeons to balance their commitment to good patient care with intense pressures for increased efficiency and decreased case turnaround time.

The VR training underscored the risks of prioritizing efficiency and case volume over patient safety and procedural integrity. Participants recognized how a results-driven culture, where speed and throughput are emphasized over safe surgical practice, can lead to critical errors and compromise team effectiveness. While efficiency is a necessary component of modern surgical workflows, the VR scenarios illustrated the consequences of allowing productivity pressures to override fundamental principles of patient care. One participant stated, “many other factors—fatigue [and]the pressure from hospital administration for revenue and productivity,” when reflecting on the systemic pressures driving an outcome-based mindset. The VR module demonstrated how this tunnel vision manifests at different levels of the operating room team. One participant observed, “The resident’s main mission was ‘I gotta close so I can go to the other room.’ The surgeon was like ‘I gotta get these 12 cases done because that’s what administration says.’ The circulator was like ‘We gotta get these cases done so we can all go home.’” Participants also reflected on the human cost of this approach, not just for the patients but for the surgical teams themselves. One particularly striking insight was, “ ...it’s fine to be efficient. It’s not fine to be in a hurry. And I think that it’s really, really important for all of us to think about....” The training allowed participants to experience the tension between efficiency and quality care, a concept that is ubiquitous among health care settings. By highlighting the potential dangers of an outcome-driven approach, the VR module reinforced the need for deliberate, methodical teamwork, where safety is never sacrificed in the name of speed or the bottom line.

The VR training also reinforced the critical importance of attention to detail in the operating room, highlighting how even small lapses in accuracy can lead to significant consequences. By immersing participants in real-world scenarios, the module demonstrated how factors such as fatigue, communication breakdowns, and time pressures can contribute to oversights. It emphasized that attention to detail is not just an individual responsibility but a collective effort, where every team member plays a role in maintaining surgical precision.

The training reinforced the critical role of accuracy in the operating room, emphasizing how seemingly minor lapses in attention to detail can have significant consequences for patient outcomes. Participants recognized that surgical safety is not solely dependent on technical skill but also on the thoroughness of preoperative preparation, intraoperative vigilance, and collective situational awareness. Several interviewees pointed out the dangers of neglecting critical details in the operating room. For example, many participants highlighted that the failure to properly assess a patient’s anticoagulation status before surgery leads to cancellation of cases and delays in care. Another key theme was the lack of a shared mental model among the surgical team, leading to fragmented awareness of the patient’s condition. As one participant described, “I’m not sure that there was a complete understanding of the entire situation... that global shared mental model of where everybody was, the implications of the decisions, and how things were happening was kind of missing.” The scenarios also illustrated that once the ball was set in motion, no one had the care or will to change it. One participant noted, “It seemed like both the surgeon and the resident didn’t have a good sense of who the patient was, what the case was, if they were on anticoagulation. And then even when the resident realized and brought it up to the attending, the attending was like, ‘It’s fine, it’s too late, moving on.’” Ultimately, the VR experience reinforced the necessity of meticulous preparation, comprehensive team awareness, and an environment where concerns are acknowledged rather than dismissed. It demonstrated that attention to detail is not merely an individual responsibility but a collective effort, where every member of the surgical team plays a vital role in ensuring safe and effective patient care.

### Comparison With Prior Work

This pilot study is the first to quantify the effects of an immersive VR-based educational intervention focused on improving TeamSTEPPS-related behaviors among surgeons in the operating room. Overall, the findings align with previous non–VR-based research highlighting the importance of teamwork training for enhancing soft skills critical to patient safety [23-27]. Our findings also align with the conclusions of the work by Abelson et al [11] that supports the motion that VR is a feasible solution for team-based training, and Gasteiger et al [12] that postulate that technical and nontechnical skills training programs using VR for health care staff may trigger perceptions of realism and deep immersion and enable easier visualization, interactivity, enhanced skills, and repeated practice in a safe environment, which in turn may improve skills and increase learning, knowledge, and learner satisfaction. Notably, prior work using VR to teach TeamSTEPPS for cesarean section surgery showed that the VR-based content improved teamwork competencies in interprofessional surgical teams [28]. By addressing the need for teamwork training, while using the TeamSTEPPS framework, and incorporating innovative educational technologies such as VR, this study demonstrated how collaboration among surgical team members can be enhanced [28]. Finally, in a randomized trial, Liaw et al [13] showed that learning outcomes did not show an inferiority of team training using VR when compared with live simulations, which supports the potential use of VR to substitute conventional simulations for communication team training.

However, many of these initiatives concentrate on skills pertinent to the immediate context of errors, such as communication and teamwork in the operating room, as well as technical competencies, while often neglecting training related to systemic flaws, the culture of patient safety, and the unreliable thought processes and behaviors that can lead to mistakes or hinder their prevention. We propose that trainees’ comprehension of the factors leading to patient safety incidents as highlighted by the HFACS framework, their sense-making of safety behaviors as outlined by the TENTS tool, and their understanding of TeamSTEPPS principles in the context of the surgical error can lead to improvements in patient safety.

### Limitations

While the findings of this study offer valuable insights, there are several important limitations to consider. First, the results are based on a single experiment with a relatively small sample size from 1 academic medical center. To address the small sample size, we used a 2-tailed, ANOVA for significance between the groupings using the FDR to adjust the resulting *P* values (from the 2-sample *t*-tests) to account for the multiplicity of the tests. For a 2-tailed, ANOVA for significance between the groupings, without the FDR control, the analysis would require approximately 68 participants to obtain a medium and to large effect size, the power level of 0.8, and an α of .05. Larger-scale studies could also take into account various confounding factors, such as training levels, gender, and race. Additionally, the possibility that more motivated individuals enrolled in the intervention group may have skewed the results. Second, the participants’ awareness of being observed may have influenced their performance, potentially biasing the results toward better patient safety practices. To mitigate this effect, all participants were allowed to withdraw from the study at any time and were assured that their individual results would remain confidential. Third, the TENTS instrument and scoring could be imperfect. We address this by conducting robust training and practice with the TENTS tool and by blinding students to the treatment status of the surgeons during postintervention observations. Despite the limitations associated with this approach, the involvement of medical student volunteers was essential for executing this extensive observational study without incurring financial costs. Future iterations of this research could leverage such a program, benefiting both the institution through enhanced understanding of behaviors in operating rooms and the students through valuable operating room exposure and hospital experience. In summary, while the findings offer valuable insights, the limitations of this single-site study with a small sample size and potential participant bias must be considered. Larger, more robust studies will be needed to validate and expand upon these preliminary results. There is also a need for longitudinal studies to assess effects over time.

### Conclusions

A VR-based immersive training program focused on TeamSTEPPS principles appears effective at improving safety behaviors, as measured by the TENTS tool. Our qualitative analysis based on 492 quotes from participants, suggest that the observed behavioral changes are a result of an immersive experience and sense-making of key training concepts where participants could see the consequences of suboptimal teamwork (eg, poor leadership and communication, lack of attention to detail, failure to take responsibility, low psychological safety to speak up, etc). Given the persistent patient safety issues in operating rooms nationwide, such innovative and immersive patient safety education programs could provide a scalable intervention to help reduce patient harm in the long run. However, further research with larger, more diverse sample sizes is needed to confirm the generalizability of these findings.

## Supplementary material

10.2196/66186 Multimedia Appendix 1 One-minute overview clip of our VR training used in this study. VR: virtual reality.

## Abbreviations

FDR: false discovery rate
HFACS: Human Factors Analysis and Classification System
TeamSTEPPS: Team Strategies and Tools to Enhance Performance and Patient Safety
TENTS: Teamwork Evaluation of Non-Technical Skills
VR: virtual reality
